# Hypothyroidism as a risk factor for open angle glaucoma: A systematic review and meta-analysis

**DOI:** 10.1371/journal.pone.0186634

**Published:** 2017-10-25

**Authors:** Shiming Wang, Yue Liu, Guangying Zheng

**Affiliations:** 1 Department of Ophthalmology, the First Affiliated Hospital of Zhengzhou University, Zhengzhou, China; 2 Aier Eye Hospital Group, Chongqing Aier Eye Hospital, Chongqing, China; Oregon Health and Science University, UNITED STATES

## Abstract

**Purpose:**

The relationship between hypothyroidism and primary open angle glaucoma (POAG) has attracted intense interest recently, but the reported results have been controversial. This meta-analysis was carried out to determine the association between hypothyroidism and POAG.

**Methods:**

The literature was identified from three databases (Web of Science, Embase, and PubMed). The meta-analyses were performed using random-effects models, with results reported as adjusted odds ratios (ORs) with 95% confidence intervals (CI 95%).

**Results:**

A total of 11 studies meeting the inclusion criteria were included in the final meta-analysis. The pooled OR based on 11 risk estimates showed a statistically significant increased risk of POAG prevalence among individuals with hypothyroidism (OR = 1.64, 95% CI = 1.27–2.13). Substantial heterogeneity among these studies was detected (*P* < 0.001; I^2^ = 83.2%). Sub-group analysis revealed that the cohort studies and case–control studies showed a significant association between hypothyroidism and POAG, which was not observed in cross-sectional studies. There was no significant publication bias in this study.

**Conclusions:**

The findings of this meta-analysis indicate that individuals with hypothyroidism have an increased risk of developing POAG.

## Introduction

Glaucoma, which is the major cause of irreversible blindness worldwide[1], is a complex disorder characterized by progressive optic neuropathy and corresponding visual field defects[2]. Primary open angle glaucoma (POAG) is the most common form of glaucoma. Nevertheless, the exact mechanism for POAG remains poorly understood, and a great number of patients with POAG have not been diagnosed. Recently, a large number of population-based studies have identified several risk factors associated with POAG, including diabetes[3,4], current alcohol consumption[4], myopia[5], older age[5], African ancestry[6], and a family history of POAG[7].

Hypothyroidism, which affects nearly 10% of the general population and occurs more frequently among those of advanced age, is a prevalent endocrine disease[8] characterized by low serum levels of thyroid hormones[9]. Hypothyroidism results in a reduction of cellular metabolism and yields a wide range of overt and subclinical symptoms and signs[10]. It has been hypothesized that the deposition of mucopolysaccharides in the trabecular meshwork in patients with hypothyroidism might result in rising intraocular pressure (IOP). In addition, Smith et al.[11] also found that the treatment of hypothyroidism alone would significantly improve the facility of outflow. Thus, POAG might have a significant association with hypothyroidism. In fact, several epidemiological studies have demonstrated this relationship[12,13,14].

However, there have been contradictory reports in the literature about whether hypothyroidism is independently associated with an increased risk of developing POAG. Several previously published studies have reported significant associations. Kim et al.[15] and Girkin et al.[16] found that subjects with hypothyroidism had a significantly greater risk of developing glaucoma compared with controls. Nevertheless, other studies do not support this effect[12,17,18].

Since this question is unlikely to be answered through an individual epidemiological study, the main aim of this study was to determine the potential relationship between hypothyroidism and POAG by using a systematic review and meta-analysis.

## Methods

### Search strategy

Three databases were used to conduct a computerized search of terms from their inception to February 01, 2017: Web of Science, Embase, and PubMed. The following terms were used in the search: hypothyroidism, thyroid disease, thyroid disorder, thyropathy, glaucoma, ocular hypertension, intraocular pressure, and intraocular hypertension. No language restrictions were applied. A hand search for other potential articles was carried out by checking the reference lists of the original paper.

### Inclusion and exclusion criteria

The inclusion criteria for research included in this meta-analysis were as follows: (1) cross-sectional, case–control, or cohort design, (2) evaluated the relationship between hypothyroidism and POAG, and (3) reported relative risk (RR) or odds ratios (ORs) estimates with 95% confidence intervals (CIs) (or provided sufficient data to calculate them). The exclusion criteria were the following: (1) studies involving angle-closure glaucoma or secondary glaucoma, (2) the data provided could not be used to calculate the RRs or ORs, or (3) case reports, abstracts, reviews, and reports with incomplete data. If different publications from the same study subjects were available, the most recent one was included.

### Data extraction and quality assessment

Two reviewers extracted the following information independently: first author, year of publication, study design, country of the subjects studied, methods used to determine hypothyroidism, definition of glaucoma, age of the study participants, sample size, outcomes measured with 95% CIs, and the adjusted variables. Two reviewers independently assessed study quality using the tool described by Sanderson et al.[19] The variables of the methods used for selecting study subjects, the methods used for measuring outcomes and exposure, the methods used to control for confounding, design-specific sources of bias, potential conflicts of interest, and statistical methods were examined. Any discrepancies were resolved by discussion.

### Statistical analyses

This meta-analysis was conducted using Stata 12.0 (Stata Corp., College Station, TX). Fully adjusted summary estimates were used to assess the relationship between hypothyroidism and POAG; moreover, the adjusted summary estimates and their corresponding 95% CIs were pooled using the random-effects model. A homogeneity test across studies was performed using a Cochran’s Q test and the I^2^ index. For the Q statistic, *P* < 0.05 indicated statistically significant heterogeneity. For the I^2^ statistic, if I^2^ > 50%, heterogeneity was said to exist[20]. Subgroup analysis according to study design, geographical area, methods used to determine hypothyroidism, publication year, and the number of adjusted variables was performed to examine the impact of these factors on the association. Sensitivity analyses were also conducted, by which the influence of a single study on the pooled effect was examined by removing one study at a time. Potential publication bias was investigated by Begger’s funnel plots and Egger’s regression test[21,22]. *P* < 0.05 was considered statistically significant in the test for overall effect.

## Results

### Literature search

**Fig 1:**
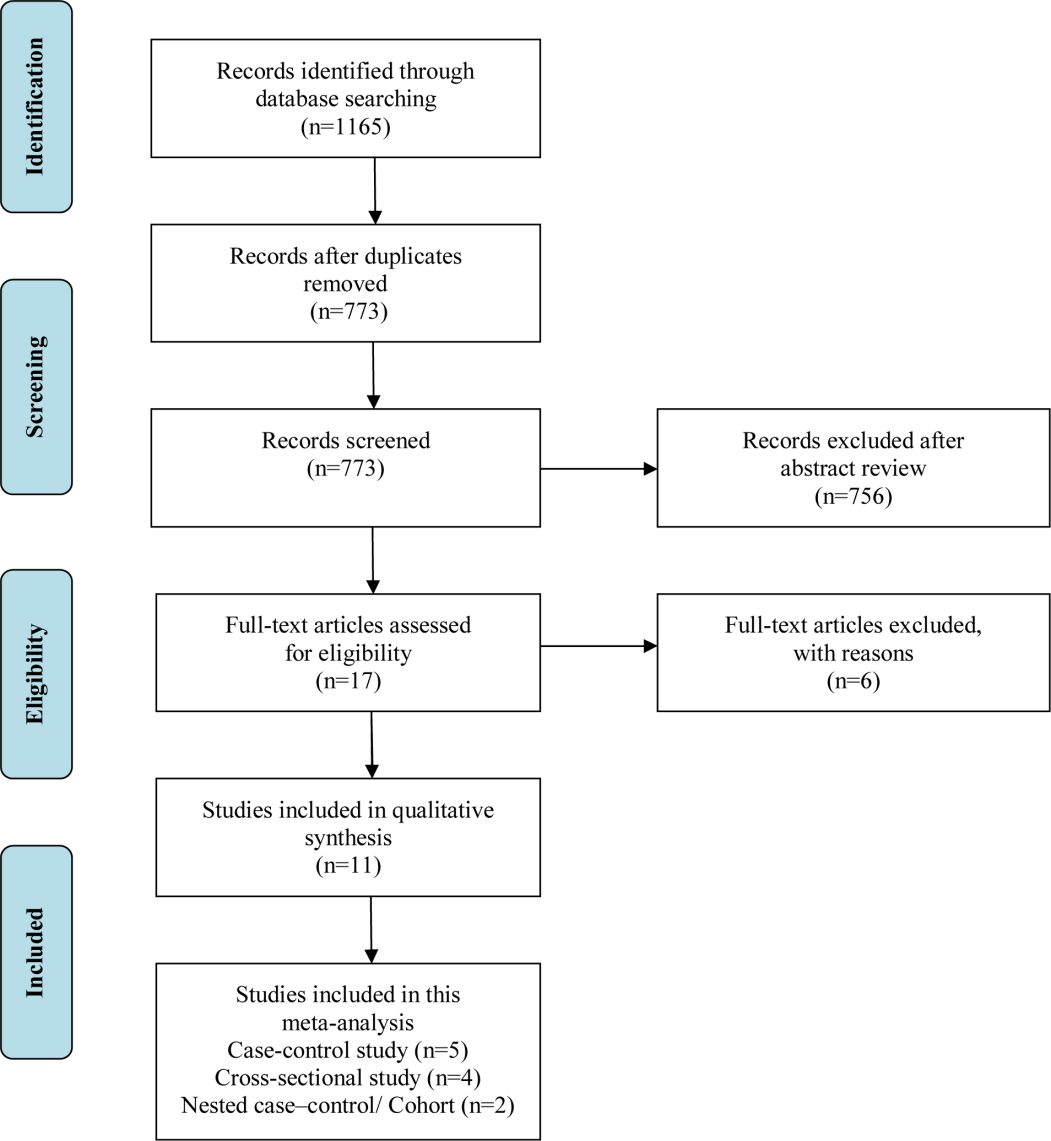
Flow diagram outlining the selection process for the inclusion of the studies in the systematic review and meta-analysis.

The initial database search yielded 1,165 potential entries, of which 392 were duplicate publications and thus removed. Of the 773 remaining publications that qualified for title and abstract review, 756 were excluded because of apparent irrelevance, e.g., non-human studies, reviews, or topics other than hypothyroidism. Consequently, 17 studies remained for further assessment and a full-text review. Among them, three studies were excluded because they did not provide sufficient data to calculate the OR[23,24,25]. Another three studies were omitted for various reasons: one only provided data about the association between hypothyroidism and chronic rhinosinusitis[26], one was not based on hypothyroidism but thyroid disease[27], and one only provided data about the association between hypothyroidism and obstructive sleep apnea[28]. Ultimately, 11 studies, including five case-control, two cohort or nested case-control, and four cross-sectional studies, met the inclusion criteria and were included in this meta-analysis [12,13,14,15,16,17,18,29,30,31,32]. The detailed process of data selection is described in Fig 1.

### Characteristics of studies and quality assessment

The characteristics of the included epidemiological studies are shown in Table 1. The 11 studies were published between 1993 and 2015 and were conducted in several different countries: Canada, Spain, Australia, the United States, Portugal, Chinese Taiwan, and Korea. Of them, five were case–control studies, four were cross-sectional studies, and two were cohort or nested case–control studies. Sample sizes in these studies ranged from 128 to 306,692. The method of definition of glaucoma varied across studies. Most of the studies defined POAG according to glaucomatous optic neuropathy and glaucomatous visual field loss. Several studies also included other diagnostic factors, such as elevated IOP, open angle, and the exclusion of secondary or angle-closure glaucoma. Most of the studies adjusted for confounders such as age, sex, and diabetes in the multivariate analysis. The detailed quality assessment of the included studies is showed in Table 2.

**Table 1:** Descriptive characteristics of studies included in the meta-analysis.

Author (year)	Location	Design	Hypothyroidism ascertainment	Definition of glaucoma	Age (case/control or Exposed /Comparison)	Sample size	Adjusted OR (95%CI)	Adjusted variables
Smith (1993)	Canada	Case–control	Medical records	GON, GVFL, open angle, exclude second glaucoma	72/70	128	5.00 (1.52–16.44)	Age, sex
Munoz-Negrete (2000)	Spain	Case–control	Blood TSH, FT4 level	GON, GVFL and/or elevated IOP	63.56/60.47	150	0.33 (0.04–3.13)	Age, sex
Lee (2004)	Australian	Cross–sectional	Self-report	GON, GVFL, exclude angle closure, second glaucoma	NA	3383	1.2 (0.5–2.9)	Age, sex, family history of glaucoma, myopia, diabetes, hypertension, pseudoexfoliation
Girkin (2004)	United States	Nested case–control	Medical records	GON, GVFL, open angle, exclude second glaucoma	69/69	6487	1.85 (1.09–3.11)	Age, sex, diabetes, lipid metabolism disorders, hypertension, cardiovascular disease, cerebrovascular disease, arterial disease, migraines.
Motsko (2008)	Portugal	Case–control	Medical records	GON, GVFL	73.6/73.5	18912	1.03 (0.90–1.17)	Age, sex, ischemic heart disease, cerebrovascular disease, hyperlipidemia, hypertension, arterial disease, diabetes, migraines.
Lin (2010)	Taiwan	Case–control	Medical records	GON, GVFL, elevated IOP, exclude angle closure	62.7/62.7	306692	1.70 (1.61–1.80)	Age, sex, monthly income, level of urbanization of the community
Lin (2010)	Taiwan	Cohort	Medical records	GON, GVFL, elevated IOP, exclude angle closure	67.9/69.6	2313	1.78 (1.04–3.06)	Age, sex, monthly income, hyperlipidemia, hypertension, diabetes, ischemic heart disease, cerebrovascular disease, arterial disease, migraine, urbanization level
Kim (2012)	Korea	Cross–sectional	Self-report	GON, GVFL	68.4/63.5	1464	8.39 (1.54–45.69)	Age, sex, diabetes, hypertension, family history of glaucoma
Chung (2014)	United States	Case–control	NA	GON, GVFL	56.6/56.5	28252	1.84 (1.34–2.55)	Age, sex
Shim (2015)	Korea	Cross–sectional	Self-report	GON, GVFL, open angle, exclude second glaucoma	56.38/56.24	315	4.91 (0.20–121.46)	Age, sex
Kakigi (2015)	United States	cross–sectional	Self-report	NA	61.5/57	13599	1.60 (0.87–2.95)	Age, sex, race, hypertension, BMI, annual household income, education level, smoking, alcohol intake

OR: odds ratio; CI: confidence interval; GON: glaucomatous optic neuropathy; GVFL: glaucomatous visual field loss; IOP: intraocular pressure; BMI: body mass index

**Table 2:** Assessment of Methodological Quality of Included Studies on Association between hypothyroidism and POAG.

Study	Methods for Selecting Study Participants	Methods for mearsuring exposure (Hypothyroidism)	Methods for mearsuring outcome (POAG)	Design-Specific Sources of Bias	Methods for Controlling Confounding	Conflict of Interest
Smith (1993)	Total of 64 consecutive patients presenting to the glaucoma clinic and 64 consecutive patients presenting to the general eye clinic were enrolled.	Measurement of TSH	IOP≥21mmHg with associated disc damage confirmed by visual field	Selection bias, residual confounding, chance finding, small sample size	Age, sex	None reported
Munoz-Negrete (2000)	Total of 75 consecutive patients with POAG and 75 patients as control from the general unit of the Ophthalmology Department after excluding glaucoma were enrolled.	Measurement of TSH	IOP≥21 mm Hg with visual field and/or optic nerve head damage, open angle, excluding pseudoexfoliation, pigment dispersion, and other secondary glaucomas	Selection bias, residual confounding, chance finding, small sample size	Age, sex	None reported
Lee (2004)	The Blue Mountains Eye Study examined 3654 persons who were aged 49–97 years of age.	Self-reported history of diagnosis and treatment for thyroid disease.	Visual field loss matched optic disc rim loss, excluding angle closure, rubeosis, secondary glaucoma, and pseudoexfoliation	Selection bias, chance finding, residual confounding	Multivariate analysis adjusted for age, sex, family history of glaucoma, myopia, diabetes, hypertension, and pseudoexfoliation.	None reported
Girkin (2004)	Total of 590 glaucoma patients and 5897 controls were randomly selected from the study population who did not have a glaucoma diagnosis by the end of the observation period.	Hypothyroidism cases were identified based on TSH and/or use of thyroid replacement therapy	IOP≥21mmHg with associated disc damage confirmed by visual field	Selection bias, residual confounding, chance finding	Multivariate analysis adjusted for age, sex, diabetes, lipid metabolism disorders, hypertension, cardiovascular disease, arterial disease, migraines.	None reported
Motsko (2008)	A total of 4728 newly diagnosed POAG patients were matched with 14184 controls were enrolled.	Hypothyroidism cases were identified based on TSH and/or use of thyroid replacement therapy	IOP≥21mmHg with associated disc damage confirmed by visual field	Selection bias, residual confounding, chance finding	Multivariate analysis adjusted for age, sex, ischemic heart disease, cerebrovascular disease, hyperlipidemia, hypertension, arterial disease, diabetes, migraines.	None reported
Lin (2010)	The data used in this study were sourced from the National Health Insurance Research Database	Medical records	IOP≥21mmHg with associated disc damage confirmed by visual field	Selection bias, residual confounding, chance finding	Multivariate analysis adjusted for age, sex, monthly income, level of urbanization of the community	None reported
Lin (2010)	The data used in this study were sourced from the National Health Insurance Research Database	Medical records	IOP≥21mmHg with associated disc damage confirmed by visual field	Residual confounding, chance finding	Multivariate analysis adjusted for age, sex, monthly income, hyperlipidemia, hypertension, diabetes, ischemic heart disease, cerebrovascular disease, arterial disease, migraine,	None reported
Kim (2012)	Local 2027 residents aged 40 years or older were selected for the study. 1532 received eye examinations, corresponding to an overall response rate of 79.5%.	Interviewed and completed a questionnaire	IOP≥21mmHg with associated disc damage confirmed by visual field layer, open angle	Selection bias, residual confounding, chance finding	Multivariate analysis adjusted for age, sex, diabetes, hypertension, family history of glaucoma	None reported
Chung (2014)	Total of 7063 subjects >18 years old who had received a first-time diagnosis of diagnosis of POAG were enrolled. As for the selection of controls, 21189 subjects were selected from the LHID2000.	None reported	IOP ≥ 21 mm Hg with visual field and/or optic nerve head damage	Selection bias, residual confounding, chance finding	Age, sex	None reported
Shim (2015)	A total of 315 patients from the Department of Ophthalmology outpatient service at Kangbuk Samsung Hospital were enrolled.	self-reported	Visual field and optic disc abnormality, Open angle, excluding secondary glaucomas	Selection bias, residual confounding, chance finding, small sample size	Age, sex	None reported
Kakigi (2015)	Total of 13599 subjects in the2008 NHIS participated in the baseline examination	Measurement of TSH	Self-reported diagnosis of glaucoma	Residual confounding, chance finding	Multivariate analysis adjusted for age, sex, race, hypertension, BMI, annual household income, education level, smoking, alcohol intake	None reported

POAG: primary open angle glaucoma; TSH: thyroid-stimulating hormone; IOP: intraocular pressure; BMI: body mass index

### Pooled Estimates of the Association between hypothyroidism and POAG

**Fig 2:**
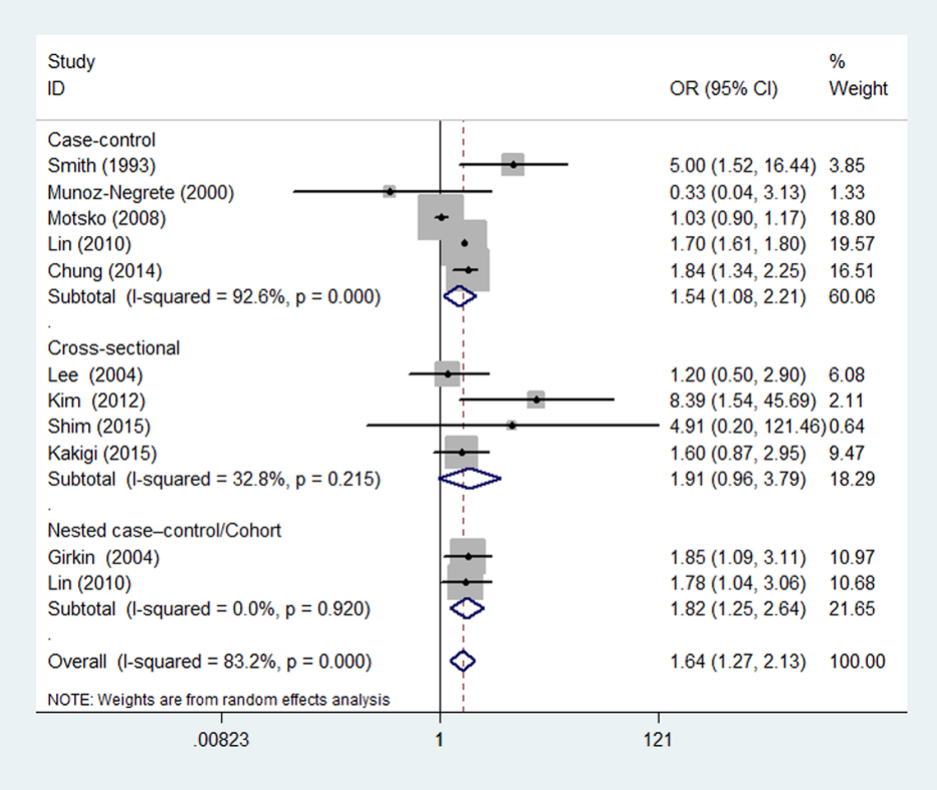
Forest plot of the risk estimates of the association between hypothyroidism and POAG.

Based on combined results calculated using the random-effect model of the 11 included studies, this meta-analysis showed that hypothyroidism might increase the risk of POAG. As shown in Fig 2, the pooled risk estimate for hypothyroidism and POAG was 1.64 (95% CI = 1.27–2.13), with significant heterogeneity (Q = 59.44; *P*_heterogeneity_ < 0.001; I^2^ = 83.2%). Considering the significant heterogeneity found among the studies, a set of subgroup analyses were performed based on study design, geographical area, methods for determining hypothyroidism, publication year, and the number of adjusted variables. In terms of stratified study design, the results showed that there was a significant positive association between hypothyroidism and POAG with a case–control design (OR = 1.54; 95% CI = 1.08–2.21; *P* = 0.018) and nested case–control/cohort design (OR = 1.82; 95% CI = 1.25–2.64; *P* = 0.002), but not in studies with a cross-sectional design (OR = 1.91; 95% CI = 0.96–3.79; *P* = 0.066). Next, subgroup analyses were performed according to geographical area. Hypothyroidism was observed to be significantly associated with POAG in studies performed in North America (OR = 1.87; 95% CI = 1.51–2.32; *P* = 0.021) and Asia (OR = 1.81; 95% CI = 1.35–2.43; *P* < 0.001). However, this association was not found in studies performed in Europe (OR = 1.00; 95% CI = 0.71–1.41; *P* = 0.991). The pooled OR was not consistent in studies where hypothyroidism status was ascertained via self-reports as well as by medical records. An association was observed between hypothyroidism and POAG in methods for determining hypothyroidism via medical records, but not in methods for determining hypothyroidism via self-reports. The impact of confounding factors on the estimate of OR was also considered. When studies adjusted for > 3 confounder factors, the summary OR showed that hypothyroidism might increase the risk of POAG; conversely, when studies adjusted for ≤ 3 confounder factors, a negative association was observed between hypothyroidism and POAG. Another subgroup analysis based on publication year was conducted, with the results showing that the 1993–2010 publication period did not significantly affect this association. In contrast, a positive association was detected between hypothyroidism and POAG in the 2010–2015 publication period. However, significant heterogeneity was still observed in some of the subgroups. Detailed information about the subgroup analysis is presented in Table 3.

**Table 3:** Subgroup meta-analyses of hypothyroidism and POAG.

		Random Effects Model		Overall Effect		Test of Homogeneity		
Subgroup	No. Studies	OR	95%CI	Z	*P*	Q	I^2^ (%)	*P*
**Study design**								
Case–control	5	1.54	1.08, 2.21	2.36	0.018	54.37	92.6	<0.001
Cross-sectional	4	1.91	0.96, 3.79	1.84	0.066	4.47	32.8	0.215
Nested case–control/ Cohort	2	1.82	1.25, 2.64	3.11	0.002	0.01	0.0	0.920
**Geographical area**								
North America	4	1.87	1.51, 2.32	5.74	<0.001	2.89	0.0	0.409
Europe	2	1.00	0.71, 1.41	0.01	0.991	1.04	4.2	0.307
Asia	4	1.81	1.35, 2.43	3.99	<0.001	3.85	22.0	0.279
**Hypothyroidism ascertainment**								
Medical records	5	1.63	1.14, 2.32	2.68	0.007	51.61	92.3	<0.001
Self-report	4	1.91	0.96, 3.79	1.84	0.066	4.47	32.8	0.215
**Adjustment for covariates**								
>3 factors	7	1.56	1.14, 2.12	2.80	0.005	52.12	88.5	<0.001
≤3 factors	4	2.09	0.89, 4.87	1.70	0.090	5.41	44.5	0.144
**Publication year**								
1993–2010	5	1.44	0.85, 2.43	1.35	0.177	12.11	67.0	0.017
2010–2015	6	1.71	1.62, 1.80	19.45	<0.001	4.21	0.0	0.519

POAG: Primary open angle glaucoma; OR: odds ratio; CI: confidence interval

### Sensitivity analysis and publication bias

**Fig 3:**
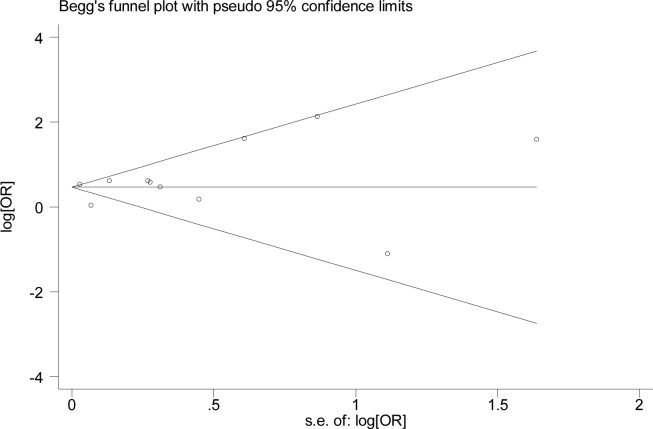
Funnel plot of the included studies evaluating the association between hypothyroidism and POAG.

To evaluate the influence of an individual study on the pooled results, a sensitivity analysis was conducted by deleting one study at a time and calculating the pooled ORs of the rest. The results showed that the deletion of any one single study did not significantly influence the change of the global estimation, suggesting the reliability and stability of the results of this meta-analysis (Table 4). In addition, the sensitivity analysis found that Motsko’s[18] and Shim’s[17] studies were the sources of the heterogeneity. After excluding Motsko’s[18] study, the pooled OR was 1.75 (95% CI = 1.54–1.99), with no evidence of heterogeneity (I^2^ = 12.0%; *P* = 0.332). After deleting Shim’s[17] study, the remaining OR was 1.63 (95% CI = 1.26–2.12), also with no evidence of heterogeneity (I^2^ = 34.7%; *P* = 0.133) (Table 4). The publication bias was evaluated by using Egger’s regression test and Begger’s funnel plots. No significant bias was found: The *P* value for Egger’s regression test was 0.612, while for Begg’s test it was 0.755, indicating a low probability of publication bias (Fig 3).

**Table 4:** Sensitivity analysis of case-control study.

	Random Effects Model		Test of Homogeneity		
Study Excluded	OR	95%CI	Q	I^2^ (%)	*P*-value
None	1.64	1.27, 2.13	59.44	83.2	<0.001
Smith (1993)	1.57	1.21, 2.04	55.88	83.9	<0.001
Munoz-Negrete (2000)	1.68	1.29, 2.18	57.44	84.3	<0.001
Lee (2004)	1.68	1.28, 2.20	59.04	84.8	<0.001
Girkin (2004)	1.62	1.23, 2.15	59.12	84.8	<0.001
Motsko (2008)	1.75	1.54, 1.99	10.23	12.0	0.332
Lin (2010)	1.68	1.19, 2.37	33.51	73.1	<0.001
Lin (2010)	1.63	1.23, 2.16	59.27	84.8	<0.001
Kim (2012)	1.59	1.22, 2.05	55.74	83.9	<0.001
Chung (2014)	1.62	1.20, 2.20	58.18	84.5	<0.001
Shim (2015)	1.63	1.26, 2.12	18.96	34.7	0.133
Kakigi (2015)	1.65	1.25, 2.18	59.44	84.9	<0.001

OR: odds ratio; CI: confidence interval

## Discussion

Although several risk factors for the development of POAG, such as diabetes[3], increased age[5], myopia[5], and family history of glaucoma[7] have been assessed, this research area remains the subject of ongoing investigation. Early on, in the 1920s, scholars had hypothesized that hypothyroidism was an important risk factor for POAG[33]. They speculated that the low metabolic condition caused by hypothyroidism would lead to decreased enzymatic activity that disadvantageously impacted aqueous humor dynamics. Thus, the belief that a certain relationship between hypothyroidism and POAG might exist has been around for some time.

In order to confirm this potential association, several epidemiological studies have tried but failed to reach a consistent conclusion. Thus, it remains controversial whether or not hypothyroidism increases the risk of POAG. Meta-analysis is a powerful statistical method that allows data on the same topic to be synthesized with greater power to obtain more accurate risk estimations. Meta-analysis was therefore conducted to reach a definite conclusion about the relationship between hypothyroidism and POAG. This study, which was based on 11 previous studies containing a total sample of 381,695 subjects, demonstrated a statistically significant association between hypothyroidism and POAG. Individuals with hypothyroidism were 1.64 times more likely to have POAG than those without hypothyroidism. Subgroup analysis showed that unlike European individuals, North American and Asian individuals had an increased risk of developing POAG. The small sample sizes and resulting insufficient statistical power of the two included studies from Europe might explain this negative association. In addition, it should be noted that in subgroup analyses of study design, cross-sectional design did not show an association; this might be explained by the significant selection bias the cross-sectional design was subject to. To achieve a reliable and convincing result, a series of analyses were carried out. Sensitivity analyses were conducted, by which the influence of a single study on the pooled effect was examined by removing one study at a time. The results showed that this analysis did not significantly change the global estimation, which supports the reliability of this study. Additionally, publication bias analysis showed a low probability of publication bias in the pooled results, further demonstrating the robustness of the present meta-analysis.

There are some possible mechanisms that could support the notion that hypothyroidism affects susceptibility to and the progression of POAG. One such mechanism is the deposition of mucopolysaccharides and hyaluronic acid in the trabecular meshwork caused by hypothyroidism, which in turn causes an obstruction in outflow that would elevate the IOP[11]. Another potential mechanism might be the increase of outflow resistance in patients with hypothyroidism. Stein et al.[34] demonstrated this by subconjunctival injection of hyaluronidase in normal and POAG subjects, from which it was determined that outflow resistance was significantly lowered in POAG patients.

This meta-analysis showed that hypothyroidism was observed to significantly increase the risk of POAG, although the substantial heterogeneity between effect size estimates limits the ability to draw firm conclusions. This heterogeneity may reflect different eligibility criteria, study populations, definitions of POAG and hypothyroidism, and/or levels of adjustment for potential confounders. To investigate the sources of heterogeneity, stratified analyses according to study design, geographical area, methods for determining hypothyroidism, publication year, and the number of adjusted variables were performed. However, only slight changes were found, and a high level of heterogeneity remained in most of the subgroups. Then, in the sensitivity procedure, it was found that heterogeneity among the included studies could be attributed to Motsko’s[18] and Shim’s[17] studies; when these studies were excluded, the remainder showed no evidence of heterogeneity. For the Shim’s study[17], several reasons may attribute to the heterogeneity. First, the relative small sample size might be the main source of heterogeneity. In this study, subjects were POAG patients with normal intraocular pressure (IOP) but not IOP ≥ 21 mm H, as other studies reported. Therefore, patient selection might be the second source of heterogeneity. Third, the low incidence of hypothyroidism in all included subjects might be another cause of heterogeneity. As for the Motsko’s study[18], the exact reasons attribute to the heterogeneity is not clear.

The major strength of this study was that it performed the first meta-analysis to assess the association between hypothyroidism and POAG via an extensive literature search that included as many relevant studies as possible to obtain a more precise conclusion. Another strength of the present study is that the enlarged sample size gave the meta-analysis enhanced statistical power to obtain a more reliable estimation of the association between hypothyroidism and POAG. Lastly, the studies included in this meta-analysis were conducted in different countries, making the results more generalizable.

Several limitations of this study should be acknowledged. First, the diagnosis of hypothyroidism in several of the studies was ascertained based on self-reports, which could cause the misclassification of hypothyroidism patients as non-hypothyroidism subjects. This underestimation might attenuate the true correlation between hypothyroidism and POAG. Second, the sample sizes in some subgroups were too small and had insufficient statistical power to reach a positive association. Third, some of the included studies failed to adjust for common confounding variables known to be risk factors for POAG, which might have affected the pooled results. Fourth, the possibility of publication bias is a major problem in any meta-analysis, because statistically significant results are more likely to be published than those with null results. Nevertheless, we found no evidence of publication bias using Begger’s and Egger’s tests in this meta-analysis. Finally, significant heterogeneity existed among the studies. Thus, the findings of this meta-analysis should be interpreted with caution.

In conclusion, the findings of this meta-analysis suggest that individuals with hypothyroidism have an increased risk of developing POAG. Given the limitations of this meta-analysis, this conclusion should be interpreted with caution. In the future, both experimental and epidemiological studies are needed to better understand the association between hypothyroidism and POAG.

## Supporting information

S1 Table PRISMA checklist item of meta-analysis. (DOC)

## References

[1] Quigley HA, Broman AT (2006) The number of people with glaucoma worldwide in 2010 and 2020. Br J Ophthalmol 90: 262–267. doi: 10.1136/bjo.2005.081224 16488940 10.1136/bjo.2005.081224 PMC1856963

[2] Foster PJ, Buhrmann R, Quigley HA, Johnson GJ (2002) The definition and classification of glaucoma in prevalence surveys. Br J Ophthalmol 86: 238–242. 11815354 10.1136/bjo.86.2.238 PMC1771026

[3] Zhou M, Wang W, Huang W, Zhang X (2014) Diabetes mellitus as a risk factor for open-angle glaucoma: a systematic review and meta-analysis. PLoS One 9: e102972 doi: 10.1371/journal.pone.0102972 25137059 10.1371/journal.pone.0102972 PMC4138056

[4] Wise LA, Rosenberg L, Radin RG, Mattox C, Yang EB, Palmer JR, et al (2011) A prospective study of diabetes, lifestyle factors, and glaucoma among African-American women. Ann Epidemiol 21: 430–439. doi: 10.1016/j.annepidem.2011.03.006 21549278 10.1016/j.annepidem.2011.03.006 PMC3091261

[5] Pan CW, Yang WY, Hu DN, Xu JG, Niu ZQ, Yuan YS, et al (2017) Longitudinal cohort study on the incidence of primary open-angle glaucoma in Bai Chinese. Am J Ophthalmol. 10.1016/j.ajo.2017.01.008 28104416

[6] Tielsch JM, Sommer A, Katz J, Royall RM, Quigley HA, Javitt J (1991) Racial variations in the prevalence of primary open-angle glaucoma. The Baltimore Eye Survey. JAMA 266: 369–374. 2056646

[7] Sun J, Zhou X, Kang Y, Yan L, Sun X, Sui H, et al (2012) Prevalence and risk factors for primary open-angle glaucoma in a rural northeast China population: a population-based survey in Bin County, Harbin. Eye (Lond) 26: 283–291. 22157917 10.1038/eye.2011.243 PMC3272184

[8] Helfand M (2004) Screening for Thyroid Disease. Rockville (MD): Agency for Healthcare Research and Quality (US). 20722119

[9] Hollowell JG, Staehling NW, Flanders WD, Hannon WH, Gunter EW, Spencer CA, et al (2002) Serum TSH, T(4), and thyroid antibodies in the United States population (1988 to 1994): National Health and Nutrition Examination Survey (NHANES III). J Clin Endocrinol Metab 87: 489–499. doi: 10.1210/jcem.87.2.8182 11836274 10.1210/jcem.87.2.8182

[10] Dedecjus M, Stasiolek M, Brzezinski J, Selmaj K, Lewinski A (2011) Thyroid hormones influence human dendritic cells' phenotype, function, and subsets distribution. Thyroid 21: 533–540. doi: 10.1089/thy.2010.0183 21190445 10.1089/thy.2010.0183

[11] Smith KD, Tevaarwerk GJ, Allen LH (1992) An ocular dynamic study supporting the hypothesis that hypothyroidism is a treatable cause of secondary open-angle glaucoma. Can J Ophthalmol 27: 341–344. 1490244

[12] Kakigi C, Kasuga T, Wang SY, Singh K, Hiratsuka Y, Murakami A, et al (2015) Hypothyroidism and Glaucoma in The United States. PLoS One 10: e133688. 10.1371/journal.pone.0133688 PMC4521841 26230664

[13] Lin HC, Chien CW, Hu CC, Ho JD (2010) Comparison of comorbid conditions between open-angle glaucoma patients and a control cohort: a case-control study. Ophthalmology 117: 2088–2095. doi: 10.1016/j.ophtha.2010.03.003 20570357 10.1016/j.ophtha.2010.03.003

[14] Lin HC, Kang JH, Jiang YD, Ho JD (2010) Hypothyroidism and the risk of developing open-angle glaucoma: a five-year population-based follow-up study. Ophthalmology 117: 1960–1966. doi: 10.1016/j.ophtha.2010.02.005 20557938 10.1016/j.ophtha.2010.02.005

[15] Kim M, Kim TW, Park KH, Kim JM (2012) Risk factors for primary open-angle glaucoma in South Korea: the Namil study. Jpn J Ophthalmol 56: 324–329. doi: 10.1007/s10384-012-0153-4 22661397 10.1007/s10384-012-0153-4

[16] Girkin CA, McGwin GJ, McNeal SF, Lee PP, Owsley C (2004) Hypothyroidism and the development of open-angle glaucoma in a male population. Ophthalmology 111: 1649–1652. doi: 10.1016/j.ophtha.2004.05.026 15350317 10.1016/j.ophtha.2004.05.026

[17] Shim SH, Kim JM, Woo HY, Shin KU, Koh JW, Park KH (2015) Association Between Platelet Function and Disc Hemorrhage in Patients With Normal-Tension Glaucoma: A Prospective Cross-Sectional Study. Am J Ophthalmol 160: 1191–1199. doi: 10.1016/j.ajo.2015.09.006 26384167 10.1016/j.ajo.2015.09.006

[18] Motsko SP, Jones JK (2008) Is there an association between hypothyroidism and open-angle glaucoma in an elderly population? An epidemiologic study. Ophthalmology 115: 1581–1584. doi: 10.1016/j.ophtha.2008.01.016 18355921 10.1016/j.ophtha.2008.01.016

[19] Sanderson S, Tatt ID, Higgins JP (2007) Tools for assessing quality and susceptibility to bias in observational studies in epidemiology: a systematic review and annotated bibliography. Int J Epidemiol 36: 666–676. doi: 10.1093/ije/dym018 17470488 10.1093/ije/dym018

[20] Zhou M, Zhang P, Xu X, Sun X (2015) The Relationship Between Aldose Reductase C106T Polymorphism and Diabetic Retinopathy: An Updated Meta-Analysis. Invest Ophthalmol Vis Sci 56: 2279–2289. doi: 10.1167/iovs.14-16279 25722213 10.1167/iovs.14-16279

[21] Egger M, Davey SG, Schneider M, Minder C (1997) Bias in meta-analysis detected by a simple, graphical test. BMJ 315: 629–634. 9310563 10.1136/bmj.315.7109.629 PMC2127453

[22] Begg CB, Mazumdar M (1994) Operating characteristics of a rank correlation test for publication bias. Biometrics 50: 1088–1101. 7786990

[23] Boles CB, Mignone U, Vadala G, Gastaldi C, Favero C, Brogliatti B (1997) Glaucoma and hypothyroidism. Acta Ophthalmol Scand Suppl: 47–48. 9589734 10.1111/j.1600-0420.1997.tb00475.x

[24] Gillow JT, Shah P, O'Neill EC (1997) Primary open angle glaucoma and hypothyroidism: chance or true association? Eye (Lond) 11 (Pt 1): 113–114. 9246288 10.1038/eye.1997.22

[25] Karadimas P, Bouzas EA, Topouzis F, Koutras DA, Mastorakos G (2001) Hypothyroidism and glaucoma. A study of 100 hypothyroid patients. Am J Ophthalmol 131: 126–128. 11162988 10.1016/s0002-9394(00)00724-8

[26] Chung SD, Lin CC, Ho JD, Ting J, Lin HC, Hu CC (2014) Increased risk of open-angle glaucoma following chronic rhinosinusitis: a population-based matched-cohort study. Eye (Lond) 28: 225–230. 24263380 10.1038/eye.2013.235 PMC3930259

[27] Hewitt AW, Wu J, Green CM, Lai T, Kearns LS, Craig JE, et al (2010) Systemic disease associations of familial and sporadic glaucoma: the Glaucoma Inheritance Study in Tasmania. Acta Ophthalmol 88: 70–74. doi: 10.1111/j.1755-3768.2009.01786.x 19958295 10.1111/j.1755-3768.2009.01786.x

[28] Lin CC, Hu CC, Ho JD, Chiu HW, Lin HC (2013) Obstructive sleep apnea and increased risk of glaucoma: a population-based matched-cohort study. Ophthalmology 120: 1559–1564. doi: 10.1016/j.ophtha.2013.01.006 23601803 10.1016/j.ophtha.2013.01.006

[29] Chung SD, Lin HC, Hung SH (2014) Allergic rhinitis is associated with open-angle glaucoma: a population-based case-control study. Am J Rhinol Allergy 28: e148–e151. doi: 10.2500/ajra.2014.28.4060 25197907 10.2500/ajra.2014.28.4060

[30] Lee AJ, Rochtchina E, Wang JJ, Healey PR, Mitchell P (2004) Open-angle glaucoma and systemic thyroid disease in an older population: The Blue Mountains Eye Study. Eye (Lond) 18: 600–608. 14716330 10.1038/sj.eye.6700731

[31] Munoz-Negrete FJ, Rebolleda G, Almodovar F, Diaz B, Varela C (2000) Hypothyroidism and primary open-angle glaucoma. Ophthalmologica 214: 347–349. 10965249 10.1159/000027518

[32] Smith KD, Arthurs BP, Saheb N (1993) An association between hypothyroidism and primary open-angle glaucoma. Ophthalmology 100: 1580–1584. 8414419 10.1016/s0161-6420(93)31441-7

[33] G H (1920) Einiges uber den Augendruck und Glaukom. Klin Monatsbl Augenheilkd.

[34] Stein R, Romano A, Treister G, Bartov E (1982) Effect of subconjunctival injection of hyaluronidase on outflow resistance in normal and in open-angle glaucomatous patients. Metab Pediatr Syst Ophthalmol 6: 169–174. 7185013

